# A15 THERAPEUTIC POTENTIAL OF THE GLYCOCAGE TARGETED DELIVERY SYSTEM FOR IMPROVING INFLAMMATORY BOWEL DISEASE TREATMENT

**DOI:** 10.1093/jcag/gwae059.015

**Published:** 2025-02-10

**Authors:** W Ma, S Menzies, C Wang, J Kothandapani, H Brumer, L M Sly

**Affiliations:** The University of British Columbia, Vancouver, BC, Canada; The University of British Columbia, Vancouver, BC, Canada; The University of British Columbia, Vancouver, BC, Canada; The University of British Columbia, Vancouver, BC, Canada; The University of British Columbia, Vancouver, BC, Canada; The University of British Columbia, Vancouver, BC, Canada

## Abstract

**Background:**

Inflammatory bowel disease (IBD), encompassing Crohn’s disease (CD) and ulcerative colitis (UC), is marked by chronic inflammation along the gastrointestinal tract (GIT). IBD cases are rising globally, with Canada having one of the highest prevalance rates. Thus, there is a critical need for more effective treatments. Current IBD therapy includes orally administered small molecule anti-inflammatory drugs, such as corticosteroids and JAK inhibitors. However, their use is limited by the high doses required due to systemic uptake, and consequently, negative off-target effects. To improve delivery of these drugs to the inflamed tissue in the lower GIT for better efficacy, our GlycoCaged prodrug technology links the drug to a plant carbohydrate for targeted release by gut bacterial glycosidases at the disease site. To demonstrate proof-of-principle, we used dexamethasone (DEX), a potent corticosteroid that is not used in IBD treatment due to inherent side effects. We showed that “GlycoCaging’‘ Dex improves its efficacy (10-X lower doses required) and reduces systemic side effects as compared to free DEX in the SHIP-/- model of CD-like ileitis.

We hypothesize that Glycocaging IBD drugs will increase drug efficacy and reduce off-target effects by delivering drugs to the site of intestinal inflammation.

**Aims:**

1) Investigate the location of prodrug decaging in the GIT using SHIP-/- mouse model. 2) Evaluate GlycoCaged dexamethasone in the T cell transfer model of colitis.

**Methods:**

To delineate the location of decaging activity in vivo, SHIP+/+ and SHIP-/- mice were orally gavaged with 3 mg/kg of DEX or the molar equivalent of GlycoCaged DEX and collected sections of intestinal tissues for LC-MS/MS analysis at multiple timepoints post oral gavage. To assess the GlycoCage system in another mouse model of IBD, T cell transfer model of colitis is established by injecting (i.p) CD4^+^CD25^-^CD45RB^high^ T cells (isolated from CD45.1 mice by FACS) into age- and sex-matched Rag-/- mice. A dose titration was performed from 3mg/kg/d for 5 weeks of DEX or the molar equivalent of caged DEX to determine and compare their minimal effective doses. Treatment effects were assessed with histology, flow cytometry and multiplexed cytokine assay.

**Results:**

Free DEX is absorbed early in the proximal GIT, while the GlycoCage prodrug system protects the DEX to be released starting in the distal small intestine and cecum, closer to the inflamed sites. Caged DEX ameliorated colonic inflammation in the T cell transfer model of colitis at lower doses than free DEX.

**Conclusions:**

Our results highlight a novel microbiome-cleavable glycoconjugate drug delivery platform that has the potential to improve the therapeutic indices of diverse drugs for treating IBD. In the future, we will evaluate the efficacy of other GlycoCaged drugs.

**Funding Agencies:**

CAG, CIHRTRIANGLE

